# Protective mechanisms of medicinal plants targeting hepatic stellate cell activation and extracellular matrix deposition in liver fibrosis

**DOI:** 10.1186/s13020-014-0027-4

**Published:** 2014-12-24

**Authors:** Florent Duval, Jorge E Moreno-Cuevas, María Teresa González-Garza, Carlos Rodríguez-Montalvo, Delia Elva Cruz-Vega

**Affiliations:** Cell Therapy Department, School of Medicine, Tecnológico de Monterrey, Monterrey, NL CP 63710 Mexico; Centro de Enfermedades Hepáticas-Digestivas y Nutrición, HSJ, Monterrey, NL Mexico

## Abstract

During chronic liver injury, hepatic stellate cells (HSC) are activated and proliferate, which causes excessive extracellular matrix (ECM) deposition, leading to scar formation and fibrosis. Medicinal plants are gaining popularity as antifibrotic agents, and are often safe, cost-effective, and versatile. This review aims to describe the protective role and mechanisms of medicinal plants in the inhibition of HSC activation and ECM deposition during the pathogenesis of liver fibrosis. A systematic literature review on the anti-fibrotic mechanisms of hepatoprotective plants was performed in PubMed, which yielded articles about twelve relevant plants. Many of these plants act *via* disruption of the transforming growth factor beta 1 signaling pathway, possibly through reduction in oxidative stress. This reduction could explain the inhibition of HSC activation and reduction in ECM deposition. Medicinal plants could be a source of anti-liver fibrosis compounds.

**Electronic supplementary material:**

The online version of this article (doi:10.1186/s13020-014-0027-4) contains supplementary material, which is available to authorized users.

## Introduction

Liver fibrosis is caused by inappropriate tissue repair *via* connective tissue deposition, which results from chronic liver injuries, including those from alcohol, chronic viral hepatitis, autoimmune diseases, parasites, metabolic diseases, and toxins or other drugs [1]. When fibrosis is not controlled, it can progress into cirrhosis. Cirrhosis was previously considered to be irreversible, but some studies suggest that fibrosis and cirrhosis could be reversible [2].

Liver fibrosis is a public health problem that results in significant morbidity and mortality [3]. Hundreds of thousands of people worldwide suffer from cirrhosis, partially because of the obesity pandemic combined with the high incidence of alcohol abuse and viral hepatitis [4]. Chronic viral hepatitis (B and C), alcoholic liver disease, and nonalcoholic fatty liver disease are the three most common causes of liver cirrhosis [5]. The prevalence of chronic liver diseases is predicted to increase, partially owing to the rising prevalence of obesity and metabolic syndrome, especially in developed countries [6].

The pathogenesis of liver fibrosis is complex and varies among different kinds of hepatic injuries. Usually after acute liver damage, parenchymal cells are regenerated to replace the necrotic and apoptotic cells. This regenerative process is associated with an inflammatory response and a limited deposition of extracellular matrix (ECM). When the liver is subjected to chronic injury, the regenerative response fails and hepatocytes are replaced with abundant ECM, which is mainly composed of collagen types I, III, and IV; fibronectin; elastin; laminin; and proteoglycans [7]. Hepatic stellate cells (HSC) are the main source of ECM [8].

**Figure 1:**
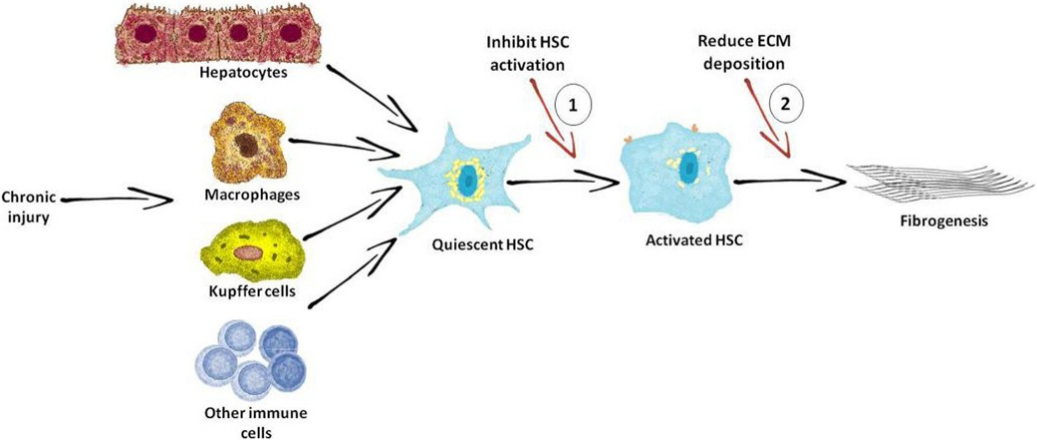
**Antifibrotic medicinal plants targeting HSC activation and ECM deposition.** HSC: hepatic stellate cells, ECM: extracellular matrix, 1*: C. longsa, S. marianum, G. biloba, S. miltiorrhiza, G. glabra, S. baicalensis, B. falcatum, Phyllanthus* species*, B. aristata, Ginseng* species*, A. paniculata,* and *Coffea* species. 2*: C. longa, S. marianum, G. biloba, S. miltiorrhiza, G. glabra, S. baicalensis, B. falcatum, Phyllanthus* species*, B. aristata, Ginseng* species*,* and *Coffea* species*.*

There is no standard treatment for liver fibrosis [7], but a reduction in liver injury events, such as cessation of alcohol intake or successful viral hepatitis treatment can control fibrosis. Nevertheless, these actions are often insufficient to avoid eventual progression to cirrhosis in the vast majority of patients [9]. Although important advances have been made in understanding the pathogenesis of hepatic fibrosis over the past 20 years, efficient antifibrotic drugs have yet to be developed. There are two ways by which medicinal plants and their bioactive compounds and extracts could reduce liver fibrosis: *via* inhibition of HSC activation and *via* reduction of ECM deposition (Figure 1). Liver fibrosis treatment should take into account the versatility of its pathogenesis and should act upon all pathways involved, beginning with HSC activation and ECM deposition.

Medicinal plants are often safe, cost-effective, and versatile, and are therefore popular potential antifibrotic agents. This review aims to describe the role of some hepatoprotective plants in the inhibition of HSC activation and ECM deposition in the pathogenesis of liver fibrosis. These plants include: *Curcuma longa, Silybum marianum, Ginkgo biloba, Salvia miltiorrhiza, Glycyrrhiza glabra, Scutellaria baicalensis, Bupleurum falcatum, Phyllanthus* species*, Berberis aristata, Ginseng* species*, Andrographis paniculata*, and *Coffea* species.

### Literature inclusion criteria

The 12 plant species were selected because of their known hepatoprotective activities. A three-step progressive searching method was applied using PubMed. In each of the steps, only pertinent articles were selected. First, a global search on the liver activity of each plant species was undertaken using the keywords “liver” and “plant species name”. The antifibrotic activities were classified under two different pathways: inhibition of HSC activation and suppression of ECM deposition. Bioactive compounds and extracts from every reviewed species were selected. Second, a more advanced search was performed using the terms “liver fibrosis” and “plant species name or bioactive extract name or bioactive compound name”. Finally, a search on the antifibrotic mechanisms of each species was performed using the terms “hepatic stellate cells activation”, “extracellular matrix”, “collagen”, and “plant species name or bioactive extract name or bioactive compound name”.

### Inhibition of hepatic stellate cell activation

#### Role of hepatic stellate cell activation in the pathogenesis of liver fibrosis

HSC activation, which includes initiation and perpetuation, is an early event in liver fibrogenesis. The activation of HSCs converts normal, quiescent vitamin A-rich cells into myofibroblast-like cells characterized by proliferation, chemotaxis, fibrogenesis, contractility, matrix degradation, retinoid loss, and white blood cell chemoattractant/cytokine release [10]-[14].

HSCs are initiated when gene expression and phenotype changes render the quiescent cells responsive to other cytokines and stimuli [10],[12]. Several paracrine stimuli from damaged hepatocytes and other neighboring cell types, such as Kupffer cells, platelets, sinusoidal endothelium, and immune cells initiate the activation of HSCs [15]. Transforming growth factor beta 1 (TGF-β1), platelet-derived growth factor (PDGF), and epidermal growth factor (EGF) mediate platelet stimuli [15],[16]. By producing fibronectin and activating latent TGF-β1, injured endothelial cells provoke HSC activation. Kupffer cells are important sources of paracrine stimuli for HSCs because they express TGF-β1, transforming growth factor alpha (TGF-α), reactive oxygen species, and lipid peroxides. These Kupffer cell paracrines lead to matrix synthesis, cell proliferation, and the release of retinoids [15] and matrix metalloproteinase (MMP)-9 [17] for collagen synthesis through the activation of latent TGF-β1 [18]. Kupffer cells also inhibit fibrogenesis *via* the production of anti-inflammatory interleukin-10 and nitric oxide (NO), which decrease collagen synthesis, increase collagenase production, and reduce cell proliferation and contractility [15]. All these stimuli trigger important changes in the composition of ECM, especially an increase in type I and III fibril-forming collagens and fibronectin [10]. This compositional transformation of the matrix induces new fibrogenic stimuli, which further exacerbate fibrosis [12].

During HSC activation, regulatory pathways like epigenetic regulation of nuclear factor kappa B (NF-κB) and reduction in peroxisome proliferator-activated receptor gamma (PPARγ) expression modulate the expression of many genes, such as type I collagen (α1 and α2 chains), smooth muscle α-actin, TGF-β1, TGF-β receptors (TβRI and II), MMP-2, and tissue inhibitor of metalloproteinases (TIMP)-1 and −2 [10],[12].

Perpetuation of HSC activation results in maintenance of the activated phenotype and therefore the generation of fibrosis [15]. Scars are formed through changes in HSC behavior, like proliferation, fibrogenesis, contractility, chemotaxis, retinoid loss, white blood cell chemoattractant/cytokine release, and matrix degradation [12].

Cell proliferation further promotes liver fibrosis by increasing the number of collagen-producing cells. Several mitogens have been identified to be responsible in liver fibrosis, namely PDGF; vascular endothelial growth factor (VEGF); thrombin and its receptor, EGF; TGF-α; keratinocyte growth factor; insulin-like growth factor; protease-activated receptor agonists; basic fibroblast growth factor; monocyte chemotactic factor; interleukin-6; connective tissue growth factor (CTGF); endothelin-1; and angiotensin-II [11],[12],[19].

HSCs migrate towards injured areas in the liver, which increases the fibrogenic response at a specific site *via* mediation of several chemoattractants, including PDGF, monocyte chemoattractant protein-1 (MCP-1), and chemokine receptor CXCR3 [10],[12].

Fibrogenesis is defined as increased fibrotic matrix production, especially that of collagen type I [12]. TGF-β1, produced by HSCs and other neighboring cell types like Kupffer cells, sinusoidal endothelial cells, bile duct epithelial cells, and hepatocytes [11], is a potent fibrogenic signal [20] as it increases the production of collagen I and other matrix constituents like fibronectin and proteoglycans [12],[21]. These effects are induced by the interaction between TGF-β1 and the membrane receptor complex formed by TβRI and TβRII, leading to the phosphorylation of intracellular mediators, namely Smad proteins. Smad proteins are divided into three groups: receptor-activated Smad (R-Smad), common-Smad (Co-Smad) and inhibitory-Smad (I-Smad). Phosphoylated R-Smad (Smad1, Smad2, Smad3, Smad5 and Smad8) dissociate from anchoring proteins and associate with Co-Smad (Smad4). R-Smad-Co-Smad complexes are transported into the nucleus where they act as transcription factors. This cascade is inhibited by I-Smad (Smad6 and Smad7) [20]. CTGF (through TGF-β-dependent and -independent pathways), endothelin-1, leptin, and angiotensin II have also been reported as fibrogenic factors for HSCs [11],[22].

Stellate cell contractility is associated with portal hypertension and may lead to the collapse and shrunken state of cirrhotic livers [23]. The increased expression of contractile proteins, smooth muscle α-actin, and smooth muscle myosins *via* calcium-dependent and calcium-independent pathways mediate the contractility of HSCs [11]. Endothelin-1, NO, angiotensinogen II, eicosanoids, atrial natriuretic peptide, somatostatin, and carbon monoxide are some of the signals that contribute to the contractile phenotype of HSCs [12]. Because HSCs are so important to fibrogenesis, the inhibition of HSC activation is a possible therapeutic approach to reversing liver fibrosis [1],[4]. For instance, PPARγ ligands [24], interferon gamma (IFNγ) [25], and antioxidants [26] reduce liver fibrosis by inhibiting TGF-β1 expression and therefore HSC activation.

#### Hepatic stellate cell activation as a target of antifibrotic medicinal plants

All the reviewed medicinal plants were shown to suppress HSC activation. Since the activation of HSCs occurs *via* a complex network of signaling pathways, different targets have been investigated to explain the antifibrotic activity (Table 1).

**Table 1:** **Inhibitory mechanisms of hepatic stellate cell activation via medicinal plants**

Medicinal plants	Bioactive compounds/extracts	Targeted fibrogenic pathways
*C. longa*[27]-[46]	Curcumin	TGF-β1^a^; CTGF^b^; PDGF^c^; TGF-α^d^; VEGF^e^; AGE^f^; leptin; LDL^g^; insulin; HIF-1α^h^; PGF^i^
*S. marianum*[47]-[54]	Silymarin	TGF-β1^a^; CTGF^b^
*G. biloba*[55]-[60]	*G. biloba* extract, EGb761	TGF-β1^a^; CTGF^b^; PAF^j^; endothelin-1
*S. miltiorrhiza*[46],[49],[61]-[100]	Water extract of *S. miltiorrhiza*; salvianolic acid A; salvianolic acid B; magnesium lithospermate B; tanshinone IIA; IH764-3	TGF-β1^a^; CTGF^b^; PDGF^c^; MCP-1^k^; endothelin-1; angiotensin II
*G. glabra*[101]-[104]	Glycyrrhizin; 18α-glycyrrhizin; glycyrrhizic acid	TGF-β1^a^
*S. baicalensis*[105]-[107]	Baicalin	TGF-β1^a^
*B. falcatum*[32],[108],[109]	Saikosaponin-A; saikosaponin-D	TGF-β1^a^
*Phyllanthus* species [110],[111]	Ethanol extract of *P. rheedii* Wight; ethanol extract of *P. niruri*	TGF-β1^a^
*B. aristata*[112]-[114]	Berberine	TGF-β1^a^
*P. notoginseng*[105],[115]-[118]	Ginsenoside Rg1; ginsenoside Rb1; *P. notoginseng* saponins; 25-OCH_3_-PPD	TGF-β1^a^; PDGF^c^
*A. paniculata*[119]	Andrographolide	TGF-β1^a^
*Coffea* species [120]-[130]	Caffeine; 1,7-dimethylxanthine; theophylline; theobromine	TGF-β1^a^; CTGF^b^; VEGF^e^; PDGF^c^

^a^Transforming growth factor beta 1, ^b^connective tissue growth factor, ^c^platelet derived growth factor, ^d^transforming growth factor alpha, ^e^vascular endothelial growth factor, ^f^advance glycation end-product, ^g^low-density lipoprotein, ^h^hypoxia-inducible factor-1α, ^i^placental growth factor, ^j^platelet activate factor, ^k^monocyte chemotactic protein-1.

Activation of TGF-β1/Smad signaling is one of the most important profibrogenic pathways [20]. The disruption of this pathway is a common feature of all of the reviewed antifibrotic plants. Down-regulation of the expression of TGF-β1 and its receptors, TβRI and II, and modulation of its mediators Smad 2, 3, and 7 has been observed *via* curcumin [28],[30],[32],[34],[37],[38] and compounds from *S. miltiorrhiza*[49],[66],[70],[75]-[78],[83],[88]-[91],[98], *G. glabra*[102],[103] and *Coffea* species [120]-[128]. Several mechanisms have been identified for the inhibition of TGF-β1 signaling *via* curcumin, like PPARγ activation and antioxidation. Curcumin inhibits NF-κB [27], leptin [39], advanced glycation end-product (AGE) [33], low-density lipoprotein (LDL) [40],[41], and insulin [42] mediated HSC activation by stimulating PPARγ activity and inducing *de novo* synthesis of glutathione [27],[33],[39] − [42]. Inhibition of NF-κB and stimulation of PPARγ activities are also observed in *G. biloba* extract [56],[57], 18α glycyrrhizin [101], glycyrrhetinic acid [104], and baicalin [107]. Salianic acid B and salvianolic acid B inhibited p38 mitogen-activated protein kinases [88],[90],[94],[99],[100] and extracellular signal-regulated kinases (ERK) signaling [46],[90],[92],[94],[96],[98],[100] by blocking phosphorylation of mitogen-activated protein kinase kinase 3/6, inhibiting expression of myocyte enhancer factor-2 [94], and suppressing phosphorylation of mitogen-activated protein kinase kinase [96],[100], in activated HSCs. Further, salvianolic acid B reduced hepatic fibrosis by disrupting angiotensin II signaling *via* down-regulation of angiotensin II receptor type 1, ERK, and c-Jun phosphorylation [98]. Andrographolide from *A. paniculata* decreased the hepatic level of cannabinoid receptor 1 *via* inactivation of c-Jun N-terminal kinases and the ERK phosphorylation cascade [119].

HSC proliferation is mediated by different proteins, including PDGF, CTGF, VEGF, and TGF-α through various signal molecules, such as ERK and focal adhesion kinase (FAK) [12]. Some plant extracts target these growth factors and their respective signaling pathways to reduce the proliferative response of HSCs. CTGF is inhibited by curcumin [34],[36], silymarin [54], *G. biloba* extract [59], *Salvia* extract [49],[76], and caffeine [120],[128]-[130]*via* inhibition of TGF-β signaling. Moreover, curcumin was shown to reduce the promoter activity of CTGF and suppress its gene expression by reducing NF-κB activity [36]. NF-κB was inhibited by suppression of ERK activity and suppression of Toll like receptor-4 gene expression *via* PPARγ activation [36].

PDGF and its receptor PDGF-βR were found to be down-regulated by curcumin [35],[37], salvianolic acid A [63] and B [92], coffee [123], and ginsenoside Rg1 [116]. Ginsenoside Rg1 down-regulated the expression of PDGF-βR by reducing NF-κB activity [116]. Different bioactive compounds from *S. miltiorrhiza* reduced HSC proliferation. The monomer IH764-3 inhibited HSC proliferation by down-regulating FAK and ERK expression [64],[65],[80], while salvianolic acid B attenuated PDGF-induced c-Jun N-terminal kinases, p38, and protein kinase C delta phosphorylations [95].

VEGF and its receptors were suppressed by curcumin [45] and coffee [122], which could also explain their amelioration of angiogenesis in the fibrotic liver. Additionally, curcumin reduced TGF-α levels [43].

Cyclins, and cyclin inhibitors, are essential proteins for the control of the cell cycle, and potential therapeutic targets for inhibiting HSC proliferation. The cyclin D1 gene was down-regulated by curcumin through PPARγ activation [30]. Inhibition of HSC proliferation by salvianolic acid A was mediated by the induction of cell cycle inhibitory proteins p21 and p27, down-regulation of cyclins D1 and E, and suppression of protein kinase B phosphorylation [63].

Medicinal plants also regulate the contraction of HSCs. For example, salvianolic acid B lowered portal pressure and attenuated the contraction of HSC by inhibiting the Ras homolog family member A signaling pathway [97] and decreasing the HSC free calcium ion concentration [93].

### Reduction in extracellular matrix deposition

#### Role of extracellular matrix deposition in the pathogenesis of liver fibrosis

Liver fibrosis is a dynamic process in which the equilibrium between ECM synthesis and degradation is impaired [11]. HSCs, neutrophils, and macrophages are the main cellular contributors to ECM degradation [1], while HSCs, portal myofibroblasts, bone-marrow-derived cells, and the epithelial–mesenchymal transition are responsible for ECM synthesis [14]. All these cells involve several molecular effectors such as matrix proteins, MMPs and TIMPs.

During fibrogenesis, several ECM proteins, especially collagens type I, III, and IV; proteoglycans; laminin; and fibronectin are over-expressed by activated HSCs and other cells, which results in pathological scar formation [131]. Accumulation of ECM not only depends on up-regulation of ECM production, but also on MMP–TIMP expression [132],[133]. In the early phases of liver injury, HSCs transiently express MMP-3 and MMP-13, which is a matrix-degrading phenotype. In the later stages of liver injury, the inhibition of fibrillar liver collagen degradation prevails over ECM synthesis, as evidenced by increased TIMP-1 expression, which leads to a decrease in collagen degradation by MMP-1 and MMP-13 [133]. TIMP-1 was found to protect HSCs from apoptosis [134]. Regulation of the plasminogen activation system, including enhanced production of the urokinase-plasminogen activator (uPA) and uPA receptor and modulation of plasminogen activator inhibitor type 1 (PAI-1), is another method by which HSCs regulate the ECM [135]-[137]. Macrophages also take part in matrix breakdown [9],[138], since macrophages produce MMPs, like MMP-13 [139],[140] and MMP-9 [141],[142] through the mitogen-activated protein kinase and NF-κB pathways [143].

The dynamic process of matrix degradation and synthesis could be a therapeutic target to reversing liver fibrosis and restoring normal liver architecture [4],[11],[144]. Indeed, MMP-8 cloned into an adenoviral vector reversed fibrosis in cirrhotic animal livers [145]. *In vivo* reduction of liver fibrosis has also been observed with similar therapeutic approaches involving MMP-1 [146], TIMP-1 scavengers [147], and uPA [148].

#### Extracellular matrix deposition as a target of antifibrotic medicinal plants

All of the reviewed medicinal plants, except *A. paniculata*, were shown to reduce ECM deposition. Plant compound targets include multiple components of the ECM, such as collagen species, laminin, and fibronectin, as well as MMPs, TIMPs, and the plasminogen activation system (Table 2).

**Table 2:** **Inhibitory mechanisms of extracellular matrix deposition via medicinal plants**

Medicinal plants	Bioactive compounds/extracts	Mechanisms of reduction in ECM deposition			
		↓ECM^a^proteins	↑MMPs^b^	↓TIMPs^c^	Plasminogen activation system
*C. longa*[6],[30],[32],[34],[36],[37],[42]-[44],[46],[149]-[159]	Curcumin	Collagen type I; collagen type III; fibronectin; hyaluronic acid; laminin	MMP-2; MMP-9; MMP-13; MMP-7	TIMP-1; TIMP-2	_
*S. marianum*[47],[48],[50],[51],[160]-[165]	Silymarin; silibinin (silybin)	Collagen type I; collagen type III	_	TIMP-1; TIMP-2	_
*G. biloba*[55]-[59],[166]-[169]	*G. biloba* extract; EGb761	Collagen type I; collagen type III; laminin; hyaluronic acid; collagen type IV	MMP-1	TIMP-1	_
*S. miltiorrhiza*[46],[49],[65],[66],[75]-[91],[94],[96],[98],[99],[170]-[179]	Salvianolic acid A; salvianolic acid B; salvianic acid A; salianic acid B; IH764-3; magnesium; lithospermate B; tanshinone IIA; SMND-309	Collagen type I; collagen type III; hyaluronic acid; collagen type IV; laminin	MMP-13	TIMP-1	**↓** PAI-1^d^; **↑** uPA^e^
*G. glabra*[102]-[104],[180],[181]	Glycyrrhizin; glycyrrhetinic acid; glycyrrhizic acid	Collagen type I; collagen type III	MMP-9	_	_
*S. baicalensis*[105],[106]	Baicalin	Collagen type I; collagen type III; hyaluronic acid collagen type IV	_	TIMP-1	_
*B. falcatum*[32],[108],[182]	Saikosaponin A; saikosaponin D	Collagen type I; hyaluronic acid; collagen type IV; laminin	_	_	_
*Phyllanthus* species [111],[183]	*P. amarus* extract; ethanol extract of *P. niruri*	Collagen type I	MMPs	TIMPs	_
*B. aristata*[114]	berberine	_	MMP-2	_	_
*P. notoginseng*[105],[115],[117],[118],[184],[185]	*P. notoginseng* saponins; ginsenoside Rb1; red ginseng extract; 25-OCH_3_-PPD	Collagen type I; collagen type III; hyaluronic acid; collagen type IV	MMP-13	TIMP-1	**↓** PAI-I^d^
*Coffea* species [122],[124]-[126],[128],[186]-[189]	Decaffeinated coffee; normal coffee; 1,7-dimethylxanthine; caffeine; chlorogenic acid	Collagen type I	_	_	_
		Collagen type III			

**↓**: inhibitory effect, **↑**: increased effect, ^a^extracellular matrix, ^b^matrix metalloproteinase, ^c^tissue inhibitor of metalloproteinase, ^d^plasminogen activator inhibitor type 1, ^e^urokinase-plasminogen activator.

Curcumin, silymarin, silybin, silibinin, *G. biloba* extracts like GbE761, salvianolic acids A and B, salianic acid B, *S. miltiorrhiza* extracts, magnesium lithospermate B, tanshinone IIA, monomer IH764-3, glycyrrhizin, glycyrrhetinic acid, glycyrrhizic acid, baicalin, saikosaponin A and D, *P. amarus* extracts, berberine, *P. notoginseng* saponins, ginsenoside Rb1, red ginseng extract, conventional and decaffeinated coffee, 1,7-dimethylxanthine, caffeine, and chlorogenic acid, reduced hepatic collagen content by down-regulating hepatic expression of type I and III collagen and/or decreasing the serum levels of type III procollagen and type IV collagen. Fibronectin expression was reduced by curcumin [37]. Serum levels of laminin and hyaluronic acid were lowered by curcumin, *G. biloba* extracts like GbE761, salvianolic acid B, SMND-309, baicalin, saikosaponin D, and *P. notoginseng* saponins. Expression of MMPs, like MMP-1, −2, −7, −9, and −13, were enhanced by curcumin [30],[43],[154],[157], *G. biloba* extract [166], *S. miltiorrhiza*[66],[77], salianic acid B [90], monomer IH764-3 [178], glycyrrhizin [181], baicalin [105], berberine [114], and *P. notoginseng* saponins [185], which stimulate the degradation of collagen deposits. However, MMPs have been observed to be downregulated after treatment with curcumin [158], silymarin [50],[51], *S. miltiorrhiza*[172], salvianolic acid B [175], ethanol extract of *P. niruri*[111], ginsenoside Rb1 [117], conventional and decaffeinated coffee, and caffeine [125],[126],[128]. Tissue remodeling induced by MMPs progresses liver fibrosis, so reducing MMP expression could be an antifibrotic strategy. Moreover, curcumin, silymarin, *G. biloba* extract, *S. miltiorrhiza*, monomer IH764-3, baicalin, *P. amarus* extract, *P. notoginseng* saponins, and ginsenoside Rb1 inhibited the expression of TIMP-1 and −2. Salvianic acid A (danshensu) and red ginseng extract down-regulated the expression of PAI-1 [173],[184], while salvianic acid A up-regulated uPA expression [173].

Since ECM proteins, MMPs, and TIMPS are over-expressed when HSCs are activated, the inhibition of HSC activation and proliferation is the main method by which plants can suppress ECM deposition. The antifibrotic mechanism is mainly the result of down-regulating the TGF-β1/Smad signaling pathway.

### Highlights

Inhibition of HSC activation and subsequent ECM deposition *via* medicinal plants is the result of TGF-β1/Smad signaling disruption. The down-regulation of TβRI and II and modulation of Smads suggests a common mechanism upstream to the pathway affected by the plants. Since oxidative stress is involved in the activation of HSCs, antioxidative properties could be the common mechanism by which the plants inhibit HSC activation and ECM deposition.

The antifibrotic properties of medicinal plants have mainly been observed in liver fibrosis models *in vitro* and *in vivo*. Clinical studies are sparse and mainly recruited chronic hepatitis B and C patients to assess the hepatoprotective effects of medicinal plants. Silymarin, glycyrrhizin, and *S. miltiorrhiza* have been somewhat successfully tested. Glycyrrhizin treatment induced a decrease in alanine transaminase and maintenance and improvement of necroinflammation in chronic hepatitis C nonresponders, and those patients unlikely to respond to interferon therapy when they receive at least three injections weekly over the course of 4, 22, or 52 weeks [190]-[193]. Since glycyrrhizin had no effect on hepatitis C RNA [190], investigation of the hepatoprotective mechanisms, such as inhibition of HSC activation and ECM deposition, is necessary.

Salvia injection and salvianolic acid B, have been tested in patients infected with hepatitis B. Salvianolic acid B, administrated over 6 months, reversed liver fibrosis and inflammation more effectively than IFNγ in patients with hepatitis B, as suggested by significant improvement in serum hyaluronic acid, laminin, type IV collagen, and procollagen III peptide as compared with the IFNγ group [194]. The same results, as well as decreased levels of alanine transaminase and aspartate transaminase were observed in fibrotic and cirrhotic patients infected with hepatitis B virus who were treated with Salvia injections over 45 [195] or 60 days [196]. However, another study on chronic hepatitis B patients treated with *S. miltiorrhiza* injection showed improvements only in symptoms, physical signs, and liver functions, not antifibrotic effects. Therefore, additional clinical studies are required to understand the effect of *S. miltiorrhiza* injection on liver fibrosis [197].

Clinical studies on silymarin administration for the treatment of hepatitis C were inconclusive. Silymarin has antiviral activity and has been associated with reduced progression from fibrosis to cirrhosis in advanced hepatitis C patients [198]. However, it has low oral bioavailability at 0.73% in rat plasma [199]-[201] and has not always been associated with hepatic improvement. For example, it was not effective in patients with chronic viral hepatitis C infection that who also unsuccessfully treated with interferon-based therapy [202].

Besides HSC activation and ECM deposition, other events like oxidative stress, inflammation, and immune responses are involved in the fibrogenic response [10],[11],[13],[14]. These pathways are potential targets that could help reduce hepatic fibrosis. Nevertheless, several medicinal plants have been shown to inhibit HSC activation and ECM deposition during liver fibrosis.

## Authors’ original submitted files for images

Below are the links to the authors’ original submitted files for images. Authors’ original file for figure 1

## Abbreviations

AGE: Advanced glycation end-product
Co-Smad: Common-Smad
CTGF: Connective tissue growth factor
ECM: Extracellular matrix
EGF: Epidermal growth factor
ERK: Extracellular signal-regulated kinases
FAK: Focal adhesion kinase
HSC: Hepatic stellate cells
IFNγ: Interferon gamma
I-Smad: Inhibitory-Smad
LDL: Low-density lipoprotein
MCP-1: Monocyte chemoattractant protein-1
MMP: Matrix metalloproteinase
NF-κB: Nuclear factor kappa B
NO: Nitric oxide
PAI-1: Plasminogen activator inhibitor type 1
PDGF: Platelet-derived growth factor
PDGF-βR: Platelet-derived growth factor beta
PPARγ: Peroxisome proliferator-activated receptor gamma
R-Smad: Receptor-activated Smad
TGF-α: Transforming growth factor alpha
TGF-β1: Transforming growth factor beta 1
TβRII: Transforming growth factor beta 1 receptor II
TβRI: Transforming growth factor beta 1 receptor I
TIMP: Tissue inhibitor of metalloproteinases
uPA: urokinase-plasminogen activator
VEGF: Vascular endothelial growth factor
