# Supplementary figures and images for: Protective mechanisms of medicinal plants targeting hepatic stellate cell activation and extracellular matrix deposition in liver fibrosis
Source: Chin Med. 2014 Dec 24;9:27. doi: 10.1186/s13020-014-0027-4 (PMC4299307; doi:10.1186/s13020-014-0027-4)

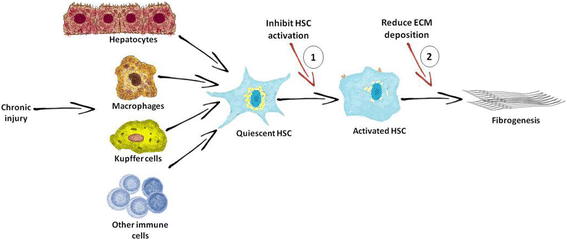

Supplement: Supplementary file 1 — Authors’ original file for figure 1 [file 13020_2014_27_MOESM1_ESM.gif]
